# Post-Deposition Wetting and Instabilities in Organic Thin Films by Supersonic Molecular Beam Deposition

**DOI:** 10.1038/s41598-018-30567-7

**Published:** 2018-08-13

**Authors:** Fabio Chiarella, Carmine Antonio Perroni, Federico Chianese, Mario Barra, Gabriella Maria De Luca, Vittorio Cataudella, Antonio Cassinese

**Affiliations:** 1 SPIN-CNR Division of Naples, Piazzale Tecchio, 80, I-80125 Naples, Italy; 2 Physics Department “Ettore Pancini”, University of Naples Federico II, Via Cinthia snc., I-80125 Naples, Italy

## Abstract

We discuss the formation and post-deposition instability of nanodrop-like structures in thin films of PDIF-CN2 (a perylene derivative) deposited via supersonic molecular beam deposition technique on highly hydrophobic substrates at room temperature. The role of the deposition rate on the characteristic lengths of the organic nanodrops has been investigated by a systematic analysis of atomic force microscope images of the thin films and through the use of the height-height correlation function. The nanodrops appear to be a metastable configuration for the freshly-deposited films. For this reason, post-deposition wetting effect has been examined with unprecedented accuracy throughout a year of experimental observations. The observed time scales, from few hours to months, are related to the growth rate, and characterize the thin films morphological reordering from three-dimensional nanodrops to a well-connected terraced film. While the interplay between adhesion and cohesion energies favors the formation of 3D-mounted structures during the growth, wetting phenomenon following the switching off of the molecular flux is found to be driven by an instability. A slow rate downhill process survives at the molecular flux shutdown and it is accompanied and maybe favored by the formation of a precursor layer composed of more lying molecules. These results are supported by simulations based on a non-linear stochastic model. The instability has been simulated, for both the growth and the post-growth evolution. To better reproduce the experimental data it is needed to introduce a surface equalizer term characterized by a relaxation time taking into account the presence of a local mechanism of molecular correlation.

## Introduction

Thin or ultra-thin film coating of a solid surface is a technological issue for a wide number of applications from flexible optoelectronics^1,2^ to adhesives^3^ and other emerging applications^4^. The study of aggregation, condensation and growth processes in the solid state continues to be of fundamental interest for applications through the control of the coating formation and its chemico-physical properties. For example, the electrical performances of an organic thin-film transistor (OTFT) is strictly related to the final morphology, the molecular assembly and the microscopic structure of the organic film which, in turn, is directly linked to various constructive parameters such as deposition technique, deposition rate or molar concentration, temperature of the substrate and the thermodynamic properties of the surface^5,6^.

In the past decades, a lot of studies have focused on the stability and self-organization of coated surfaces. Meso-structure modification induced by dewetting^7–9^ and spreading effects^10^ result ubiquitous for thin-film technology which can either be deleterious, destabilizing the thin-film structure, or advantageous, leading for example to the controlled formation of an array of nanometric islands^11^ or micro-nano-patterning^12,13^.

In the framework of vapor-deposited thin-films, for metals and inorganic semiconductors, the knowledge of the kinetic growth mechanism by vapor condensation is well assessed since the mass transport between layers is primarily ascribed to the Ehrlich-Schwöbel (ES) energy barrier. This approach gives an accurate control of the film properties and the instabilities are mainly due to the relaxation of strain elastic energy in hetero-epitaxial growth^14^. In contrast, the growth mechanism of organic thin films remains poorly understood. Several studies have tried to cover the gap, following the scaling theories^15^ and assuming that the principles ruling the inorganic growth can be universally transposed to weakly interacting systems such as organic semiconductors. It is however important to remark that the intrinsic anisotropy and the internal degree of freedom of the molecules respect to single atoms induce a series of activation barriers for crossing the steps that depend on configuration and orientation of the diffusing species^16^. This feature leads to a strong tendency to roughening (or “rapid roughening”) and to a transition from the layer-by-layer growth mode of the early stage to a 3D-mounded morphology^17–20^. Furthermore, the strongly anisotropic molecule-molecule interaction, mediated by the interaction with the solid surface, induces a multiple energetic minima in the possible molecular packing enabling polymorphs and surface induced phases^21^. We point out that the energetic non-equilibrium thermodynamic conditions of the growth do not always stabilize when the flow of molecules ceases. Post-deposition relaxation phenomena can intervene to change the film micro-structure and cracks or dewetting processes can be observed typically favored by the presence of temperature gradients or external fields. Spinodal dewetting due to capillary waves or instability^22,23^ and elastic or strain energy relaxation are the main intrinsic perturbation in solid-state dewetting of thin films^24^. Conversely, the reverse phenomena is more difficult to observe: spontaneous wetting process of a solid surface results very intriguing and unconventional in molecular thin films, even if it is common in liquids or viscous polymers^10^.

**Figure 1:**
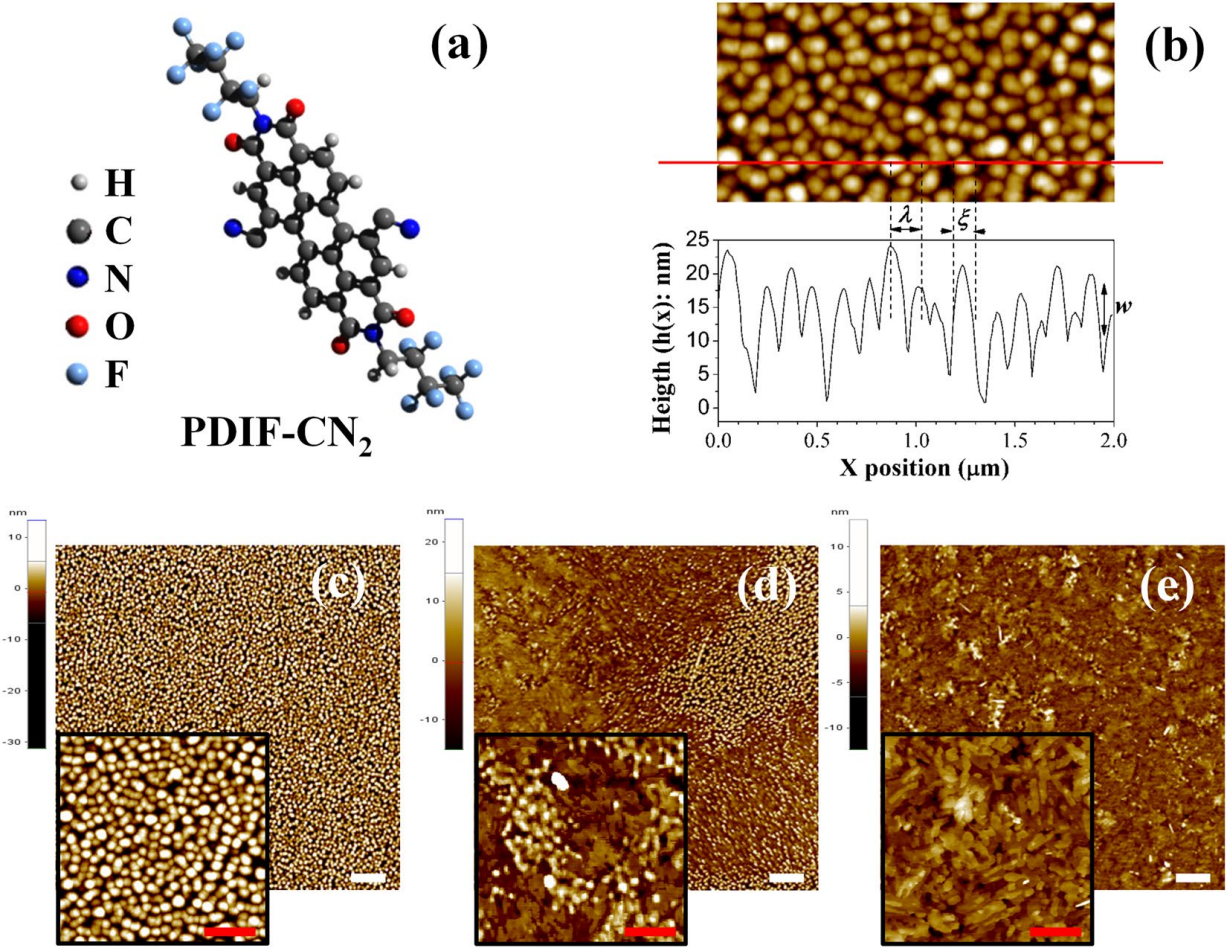
Molecular structure of PDIF-CN_2_ (**a**). The morphology of a film deposited at rate *R* = 0.07 nm/min acquired on a scan window of 2 × 1 *μ*m^2^ with the line profile acquired along the red line are shown (**b**). The characteristic length *w*, *ξ* and *λ* are depicted as illustration. In the bottom part of the image three 10 × 10 *μ*m^2^ AFM topographies are presented: the as-grown surface after few hours from the molecular beam switch off (**c**), a mixed surface (**d**) and a completely ripened surface after 1 year (**e**). The white markers are of size 1 *μ*m. In the insets, images of 2 × 2 *μ*m^2^ zoom of the images (**c**–**e**) are presented. The red markers are of size 0.5 *μ*m.

Among the broad variety of molecular compounds studied in molecular-beam-deposited thin-films, perylene derivatives alike DIP (diindenoperilene)^17,25,26^ or PTCDA (perylene-3,4,9,10-tetracarboxylic-dianhydride)^27^ have been intensively studied as prototype molecules to better understand the specific growth mechanism of extended planar molecules. PDIF-CN_2_ (N,N′-1H,1H-perfluorobutil dicyanoperylene-3,4,9,10-bis dicarboximides see Fig. 1a for a sketch) has established as n-type organic semiconductor in high performance OTFTs^28–32^. We have already discussed in ref.^33^ the fabrication of PDIF-CN_2_ n-type organic thin-film transistors (OTFTs) by supersonic molecular beam deposition (SuMBD), where high mobility devices were obtained keeping the substrate at room temperature during the organic film growth. Here SiO_2_ substrate acts both as dielectric barrier and growth surface and is made hydrophobic by HMDS (Hexamethyldisilazane) monolayer coating^34^ needful to optimize the device performances^31^. SuMBD technique has demonstrated to be a powerful approach to grow molecular films and to explore unconventional growth regimes, thanks to its specificity in decoupling the deposition rate (*R*) of the molecular flux from the impinging kinetic energy (*E*_*k*_)^35,36^. Besides, deposition at high *E*_*k*_ (in the order of electronvolts) induces a reduction of the nucleation center density (*n*)^37^. This is similar to what happens in the case of increased molecular diffusivity^38^, but without increasing the substrate temperature.

More recently, we have demonstrated that PDIF-CN_2_ molecules deposited by SuMBD show a characteristic growth regime, where the interplay between the highly *E*_*k*_ value and the highly hydrophobic substrate at room temperatures drives to a three-dimensional (Volmer-Weber-like) growth with the formation of nanodrops-like islands. We observed that the layer composed of nanometric drops were unconnected, however after a long time (i.e., from days to months) a several orders of magnitude increase in the field-effect charge carrier mobility was achieved. This was related to the observation of an unconventional spreading or ripening effect of the condensate over the time^39^. It is important to remark that, as illustrated in the control experiment discussed in ref.^39^, no dewetted growth and post-deposition spreading are observed in the case of bare (less hydrophobic) SiO_2_ substrate. The specific combination of surface hydrophobicity, molecular structure, and hyperthermal deposition conditions brings the system in a mesophase like a liquid crystal that relaxes with a spontaneous wetting process occurring over very long time scales typical of a viscous phase.

Moreover, this terraced wetting dynamics was found to depend on the initial growth rate and therefore to the as-grown density and geometrical distribution of the nanodrops. In particular, the process is negligible at deposition rate lower than 0.05 nm/min and a stable nanodrops array was formed in that case. The spreading process is clearly observed at *R* around 0.08 nm/min with a faster evolution than that observed for even higher *R*^39^.

In this paper, in order to understand the mechanism inducing this critical wetting effect, we report an accurate study of the anomalous growth and relaxation effect of PDIF-CN_2_ thin films deposited by SuMBD. We present a systematic analysis of the growth and relaxation processes by measuring statistical correlation functions of the surface morphology, just after the deposition as well as over time, of two series of films at different thicknesses (10 and 20 nm). Finally, a non-linear stochastic model based on differential equations has been used to analyze both the growth and the post-deposition relaxation dynamics focusing on the ES barrier effect.

## Results and Discussion

Using atomic force microscopy (AFM) in non-contact mode, we have investigated the morphology of our films during the time. Figure 1 shows the AFM topographies of a 10 nm-thick film 1 hour, 1 month and 1 year after the end of the deposition process. A clear morphology change is observed passing from a full nanodrop-like morphology (in Fig. 1c) to a mixed one (in Fig. 1d) and ending with a more flat and compact surface (in Fig. 1e).

The theoretical framework to describe and analyze the growth processes of molecular films is well assessed and the height-height correlation function *H*(**r**) is the most effective tool to extract the scaling exponents and establish a morphology-growth mechanism correlation^15^. Unfortunately, no general approach is actually consolidated to describe the post-deposition instability observed here. We have decided to use *H*(**r**) as the key function to extract statistical quantities and classify the film morphologies.

All the correlation functions can be calculated through the height profile *h*(**r**, *t*):

1. the interface width *w*(*t*) defined from the equation: $w(t)\equiv {[\langle |h({\bf{x}},t)-\langle h({\bf{x}},t)\rangle {|}^{2}\rangle ]}^{\frac{1}{2}}$;
2. the autocorrelation function $R({\bf{r}},t)\equiv {w}^{-2}(t) < h({\bf{r}}+{\bf{x}},t)h({\bf{x}},t) >$;
3. the height-height correlation function $H({\bf{r}},t)\equiv  < {[h({\bf{r}}+{\bf{x}},t)-h({\bf{x}},t)]}^{2} > =2{w}^{2}(t)[1-R({\bf{r}},t)]$.

At a given time *t*, it is possible to give a functional form of *H*(**r**) for a mounded surface that takes into account the presence of a periodic recurrence with wavelength *λ*, adding an oscillatory term to the exponential model commonly used for self-affine surfaces^15^ as:

1 $$H(r,t)=2{w}^{2}(t)\,[1-\exp [-{(\frac{r}{\xi (t)})}^{2\alpha }]\,{J}_{0}\,(\frac{2\pi r}{\lambda (t)})],$$

where *r* is the radial distance from a generic point of the surface, *J*_0_ is the zeroth-order Bessel function. The coefficient *α* affects the behavior of *H* for small *r* and can be related to the local fractal dimensionality of the surface. Furthermore, *w* (interface width), *λ* (recurrence wavelength) and *ξ* (correlation length) can be interpreted as the surface roughness, the average peak-to-peak distance between islands and the average diameter of the mounded islands (in our case the nanodrops as illustrated in Fig. 1b). It is common to observe a power-law behavior for these characteristic lengths as a function of the deposition time: $w(t)\sim {t}^{\beta }$, $\xi (t)\sim {t}^{\mathrm{1/}z}$ and $\lambda (t)\sim {t}^{p}$, where *β*, *z* and *p* are the scaling exponents characterizing the dynamic scaling theory^15^.

**Figure 2:**
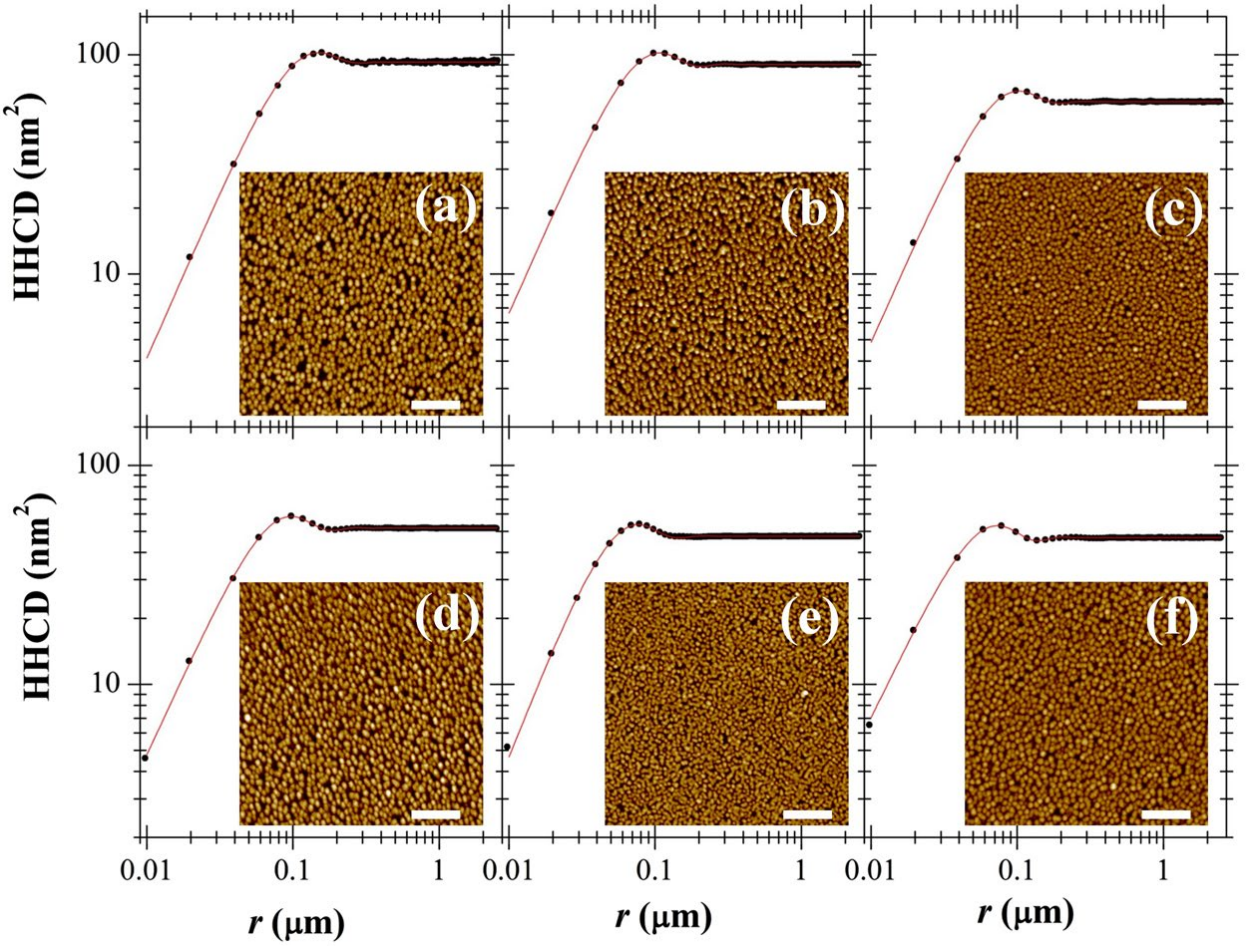
Height-height correlation data (HHCD) obtained by the 5 × 5 *μ*m AFM images of the 20-nm thick films (in the inset) via numerical calculation for the different deposition rate (from (**a**) to (**f**) *R* = 0.056, 0.074, 0.087, 0.095, 0.150, 0.218 in nm/min). the best fit curves obtained with Eq. 1 are in red (*α* = 0.7±0.1 for all the curves). The white markers is 1 *μ*m.

**Figure 3:**
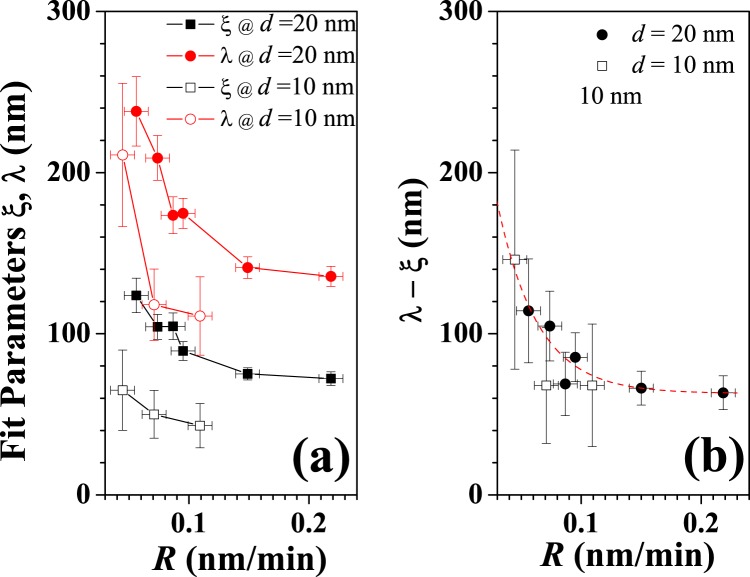
The characteristic lengths *ξ* and *λ* resulting by fitting the HHCD with Eq. 1 are reported in (**a**) for both the series of samples (*d* = 10 nm and *d* = 20 nm). The *λ* − *ξ* behaviors are plotted in (**b**).

In the framework of the dynamic scaling theory, we extract height-height correlation data (HHCD) by applying a numerical algorithm (see Gwyddion open source manual) to the AFM images (see Fig. 2) for films deposited at different rate (from 0.056 nm/min to 0.218 nm/min). The morphologies are acquired just after the flux switching off (*t* = *t*_*c*_) with a delay due to experimental timing (about 2.5 hours, i.e. 0.1 days). The HHCD are fitted with Eq. (1). From this fit we have extracted the functional dependency of the characteristic lengths on *R*. These results are shown in Fig. 3a for both the series of samples with effective thickness of 10 and 20 nm. It is important to observe the exponential behavior, where both *λ* and *ξ* rapidly decrease with *R* until to a more flat trend at rate values greater than 0.1 nm/min. A similar behavior is plotted in Fig. 3b for the difference of (*λ* − *ξ*). Assuming this quantity proportional to the average distance between the nanodrops, we get evidence that it decreases with the increase of the deposition rate without great variations changing the nominal thickness.

The obtained results can be easily discussed via geometrical evaluations considering that fixing the thickness (*d*) of the films we are fixing the deposited volume (*V*) too. Being the morphology consisting of spherical caps separated by an average distance, we can write the following equation:

2 $$d=\frac{V}{S}=\frac{AN{\xi }^{3}}{S}=An{\xi }^{3},$$

where *S* is the total surface, *A* is a geometrical factor, *N* the total number of nanodrops and *n* their density. Considering that $n\sim {(R)}^{q}$ (where *q* is a coefficient related to the dimension of the critical nucleus)^38^ and substituting it in eq. 2, a relation of proportionality between *R* and *ξ* is obtained assuming the molecular surface diffusivity fixed by the choice of the kinetic energy of the impinging molecules and the temperature of the substrate: $\xi \sim {R}^{-q\mathrm{/3}}$. Similarly, the recurrence wavelength *λ* of the nanodrop structure has a dependence from the molecular flux as $\lambda \sim {n}^{-\mathrm{1/2}}\sim {R}^{-q\mathrm{/2}}$. These considerations allow to claim that, lowering the flux at fixed film thickness, the correlation length *ξ* will increase more slowly than the *λ*, while reducing the thickness at the same deposition rate the expected *ξ* and *λ* values get reduced too. Now, being *R* = *d*/*t*_*c*_ where *t*_*c*_ is the deposition time, we can assume a dependence with *t*_*c*_: $\xi \sim {t}_{c}^{q\mathrm{/3}}$ as for Ostwald ripening^40^ and $\lambda \sim {t}_{c}^{q\mathrm{/2}}$.

**Figure 4:**
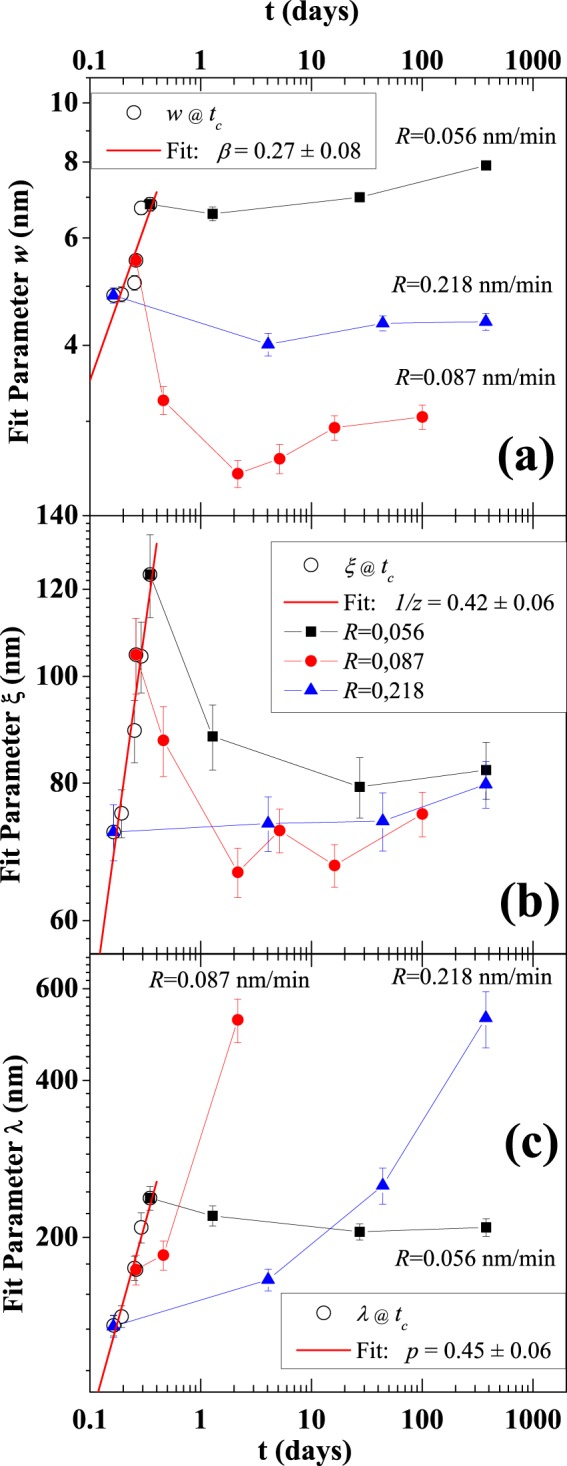
Log-log plots of the time evolution of the statistical parameters: interface width *w* (**a**), correlation length *ξ* (**b**) and recurrence wavelength *λ* (**c**) extracted by the AFM images for three 20nm-thick samples deposited at different deposition rate. The origin of the time axis coincides with the deposition starting time for all the samples. Note that the acquisition time for the as-grown morphologies are affected by a short delay Δ = 2.5 hours (i.e. 0.1 days) respect to the moment of switched off the beam (*t*_*c*_). The data obtained at about *t* = *t*_*c*_ + Δ have been used to calculate the scaling parameters by fitting procedure (the red curves).

Figure 4 shows the *ξ*, *λ* and *w* parameters extracted from the acquired images as function of the time. We have chosen three samples deposited at different deposition rate values (*R* = 0.056, 0.086, 0.218 nm/min) for which we observe a strong difference in the behavior of the wetting dynamics. The experimental data in Fig. 4 are characterized by the presence of a cusp, in correspondence of the external molecular flow ending. Indeed, when the molecular flux is active, all the characteristic parameters of the superficial morphology tend to grow with a power law, as expected in the scaling theory typically used for the description of the thin-film growth mechanism. Switching the flux off, on the other hand, these parameters relax and evolve with a different behavior, because of the system wetting relaxation effect towards a more flat surface. We find that the *ξ* values, after an initial reduction, converge to the same value over long observation times for all the different flux rates. The effect of the film micro-structure modification from nanodrops to a flat continuous film, instead, is reflected in the large increase of *λ* with a timing depending by *R* as already discussed. A peculiar behavior is observed for the roughness *w*. After an initial reduction of the value over a period of few days, the roughness increases slowly and the dynamics seems to asymptotically tend to a final value different for each starting time *t*_*c*_, i.e. for each *R* value. The observed discontinuities in Fig. 4 are, ultimately, the direct representation of the two regimes ruling the film formation with and without the presence of the external molecular flow.

From Fig. 4 (see the red lines) the scaling coefficients are obtained fitting the experimental data: *β* = 0.27 ± 0.08, 1/*z* = 0.41 ± 0.06, and *p* = 0.42 ± 0.06.

**Figure 5:**
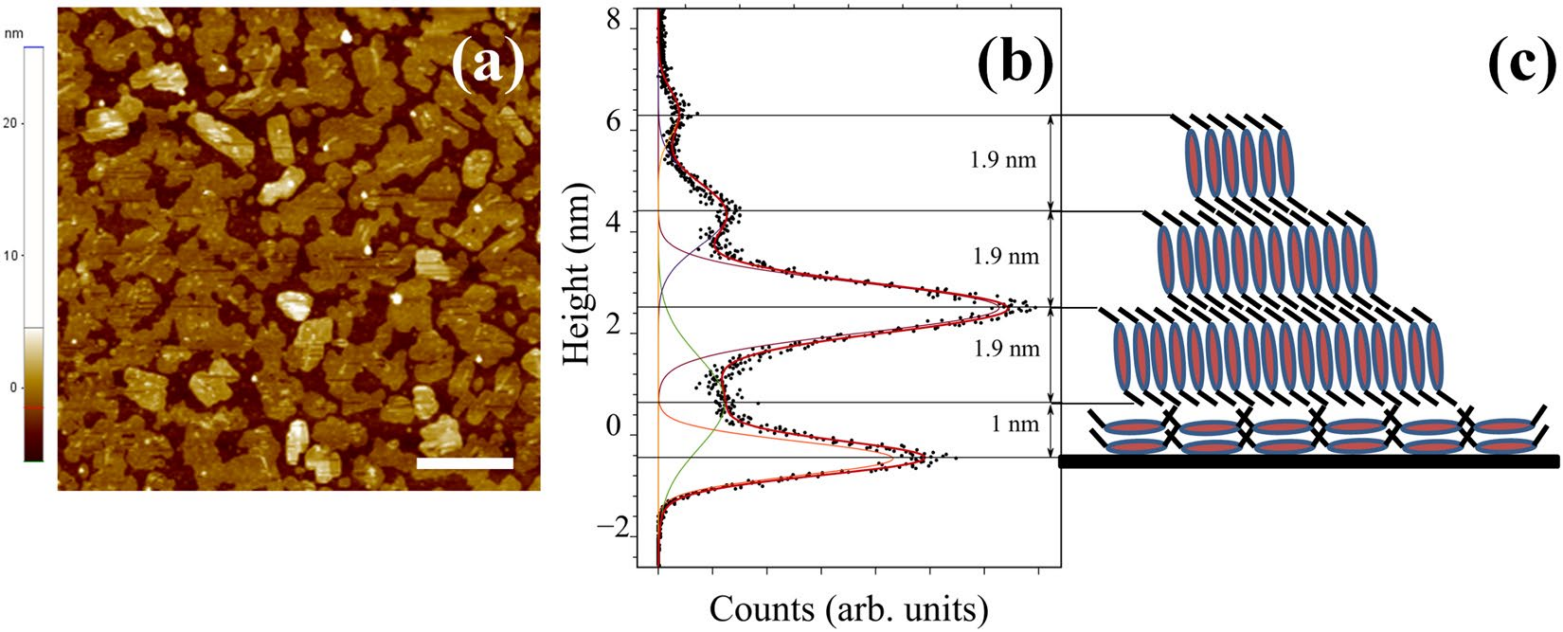
AFM image 5 × 5 *μ*m^2^ of the evolved morphology of a 3nm-thick film deposited at *R* = 0.04 nm/min (**a**) and the relative height distribution (**b**). In red we draw the sum of the Gaussian fits (thin lines) of the peaks related to the uncovered substrate and the molecular layers. In (**c**) we propose a sketch of the molecular assembly at the end of the spreading process for the 3 nm-thick film. This sketch is a suggestion just for representation. The white line is a marker of size 1 *μ*m.

To complete our experimental observation, we show in Fig. 5a the AFM image of an ultra-thin film of about 3 nm thick acquired after a long time from the end of the deposition process. Here the formation of an uncompleted layer with terraced islands gives us the opportunity to analyze the dynamics effect on the molecular system in proximity of the substrate. In Fig. 5b, the height distribution of the AFM topography is shown and the average height of the terraces of (1.9 ± 0.2) nm was measured. This is compatible with the typical PDIF-CN_2_ morphology with terrace heights comparable with the c-axis length of the crystal structure of 1.96 nm^41^, obtained in thin films when the molecules are in the stand-up configuration respect to the surface^31,42,43^.

Less conventional is the anomalous distance measured between the substrate peak and the first layer peak of (1.1 ± 0.5) nm. This could be ascribed to the presence of a surface induced phase (SIP) like a polymorph or a different molecular orientation^21^. In our case, the molecules seem to be in a lying down configuration for which the molecular core is closer to the substrate, as proposed in Fig. 5c. This observation suggests that the relaxation process is accompanied by the formation of a wetting foot around the islands acting as adhesion layer on which molecules and clusters can diffuse more effectively thanks to the weakening of the effective substrate corrugation inducing with time the observed effects of coarsening and eventually terracing. The formation of the terraces and maybe a partial dewetting can justify the increase of roughness *w* and the weak increase of the correlation length *ξ* in the final part of the morphology evolution.

Furthermore, the behavior of the observed slow wetting dynamics is strongly related to the as-grown-deposited film structure and hence to the deposition rate. As showed in Fig. 3b, the average distance between the nanodrops, proportional to (*λ* − *ξ*), results to decrease with the increase of the deposition rate. Respect to the spontaneous spreading effect of the mounds observed for the samples characterized by deposition rate values exceeding 0.05 nm/min, the experimental results suggest that for $R < 0.05$ nm/min the interaction between mounds is heavily depressed by the pronounced interstitial space. We interpret this observation as due to the fact that the precursor feet expand on the surface for a distance lower than the average distance between the nanodrops. With the reduction of (*λ* − *ξ*), the wetting foot becomes able to cover all the gap between the nanodrops making faster and more efficient the wetting process (in the range 0.07–0.10 nm/min). On the other hand, when (*λ* − *ξ*) reduces more and more at increasing *R*, the reduced space between the nanodrops could hinder and quench the formation of the wetting foot making less efficient the reformation process and producing an increase in the timing as observed.

In order to quantitatively simulate by an unified model the dynamics of growth and the following wetting process, a full-time-scale continuum-equation approach has been adopted starting from the assumption that both the processes are driven by instabilities in the growth front and molecular diffusion currents. The overall dynamics is modeled by the following equation^15,44^:

3 $$\frac{\partial h}{\partial t}=-\,\nu \nabla \cdot (\frac{\nabla h}{1+|\nabla h{|}^{2}})-\,k{\nabla }^{4}h+\eta ({\bf{r}},t)+F(t)+{(\frac{\partial h}{\partial t})}_{r},$$

where the first term describes the uphill growth due to the ES effect (∇ is the gradient operator), the second one is the Mullins diffusion term modeling the overall effect of surface diffusion. We remark that in the first term there is the sign −, therefore the ES effect introduces an instability in the growth process favoring the formation of mounds. The stochastic term *η*(**r**, *t*) is present just during the growth and modeled as Gaussian noise. When the molecular flux *F*(*t*) is switched off at time *t* = *t*_*c*_ the noise is no longer active (for a more exhaustive description see the Supplementary Information (SI)).

The last term of Eq. (3) provides a characteristic relaxation time *τ*:

4 $${(\frac{\partial h}{\partial t})}_{r}=-\,\frac{[h({\bf{x}},t)-d]}{\tau },$$

where *d* is the film thickness. This relaxation term becomes important after the flux switching off and works like a surface equalizer.

The starting point of the self-consistent approach is the observation that:

5 $$\langle |\nabla h{|}^{2}\rangle ={m}^{2}(t),$$

where *m*(*t*) is the surface slope. In order to solve the stochastic equation, we retain all the temporal fluctuations of the non-linear contribution in the first term of Eq. (3), approximating its spatial fluctuations (see SI):

6 $$\nabla \cdot (\frac{\nabla h}{1+|\nabla h{|}^{2}})\simeq \frac{{\nabla }^{2}h}{[1+{m}^{2}(t)]}\mathrm{.}$$

In the simulation we focus on the thickness of *d* = 20 nm. With the parameters *ν* and *k* present in Eq. (3)(we obtain the Mullins coefficient and the diffusion coefficient in equation 3 respectively: *k* = √2*l*_0_^4^/2*t*_0_ = 1.7 × 10^−26^ cm^4^/s and *v* = √2*l*_0_^2^/*t*_0_ = 7 × 10^−16^ cm^2^/s. The value of v is compatible with typical values of diffusivity in large cluster diffusion processes at room temperature for thin film of metals as reported in^45,46^), one can obtain the length ${l}_{0}=\mathrm{1/}{q}_{0}=\sqrt{2k/\nu }$ and the time ${t}_{0}={l}_{0}^{3}/\sqrt{\nu k}=2\sqrt{2}k/{\nu }^{2}$. The quantities *l*_0_ and *t*_0_ provide a term ${D}_{0}={l}_{0}^{4}/{t}_{0}=\sqrt{2}k$, which has the same dimensions as the term *D*, which measures the strength of the deposition noise *η****(*r**, *t*). For the dynamics analyzed in this paper, we set the following values of *l*_0_ and *t*_0_ as unit length and time, respectively: *l*_0_ = 77 nm and *t*_0_ = 1.38 days. Then, the free parameters of the theory are *R* (or *t*_*c*_), *D*, given in terms of *D*_0_ and *τ*, expressed in terms of *t*_0_. For the dynamics analyzed in this paper, *D* is of the order of 0.001 − 0.01 *D*_0_, while the relaxation time *τ* is assumed to be quite large being of the order of 6 − 7 *t*_0_.

**Figure 6:**
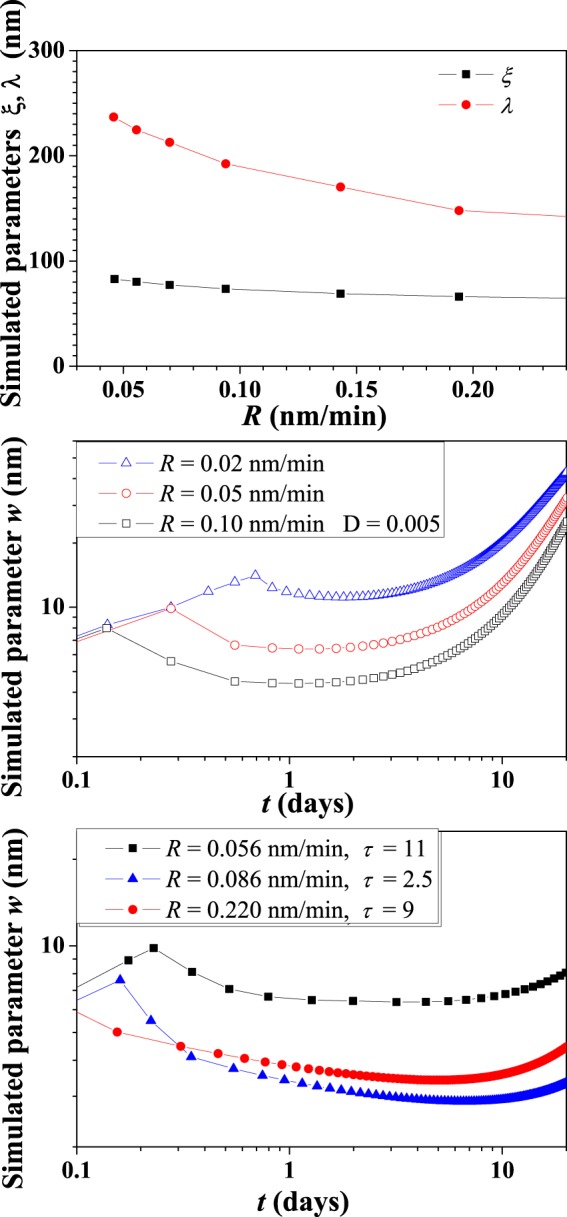
Interface width *w* (in units of nm) as a function of time *t* (in units of days) for different values of the flux rate *R* (in units of nm/min) (**a**). In the plot (**b**), simulation data for *D* = 0.005 (in units of *D*_0_), $\mathrm{1/}\tau \simeq 0$, for different *R* values. In the plot (**c**). simulation data for *D* = 0.005 (in units of *D*_0_), fixing *τ* at different value for each *R* values: *τ* = 11 at *R* = 0.056 nm/min; *τ* = 2.5 at *R* = 0.086 nm/min; *τ* = 9 at *R* = 0.220 nm/min.

Applying the model of Eq. (3) with the above-mentioned parameters, we extract the simulated characteristic lengths. In Fig. 6a, we plot the theoretical values of the correlation length *ξ* and the recurrence wavelength *λ* as a function of the flux rate, calculated at the molecular beam switch-off, in order to make a comparison with experimental data in Fig. 3a. As thoroughly discussed in the SI, the theoretical estimates for the exponents are the following: *β* = 0.291, 1/*z* = 0.405, and *p* = 0.433. The values of the exponent *β* indicate a slight deviation from the situation of dynamic scaling and a reduced instability, as found in the literature neglecting the correction *m*^2^(*t*) in the ES term of the dynamical equation (Eq. 6)^47^, (since up to times of the order of a few hours, the values of *m*^2^(*t*) are always smaller than 1, they provides weak perturbations because the denominator in Eq. (6) is close to 1 shrinking the term to the more simple, but typically used, Edwards-Wilkinson one). All these results are in good agreement with the experimental ones, suggesting that the model well simulates the unconventional hyperthermal deposition conditions.

To shed light on the interesting wetting dynamics observed after the flux switching off, we simulate the relaxation dynamics applying the model in Eq. 3 from the flux switching off up to times larger than 20 days. The theoretical approach shows that the asymptotic behavior of *ξ* at very large times is independent of the rate reaching, we can thus assume the average size of a mound as a stable quantity in the time. The *λ*, instead, increases as a function of time, reinforcing the agreement with the experiments (see images in SI).

In Fig. 6b and c, we show the results of the model concerning the less obvious behavior of the interface width *w* with time assuming $\mathrm{1/}\tau \simeq 0$ and a finite *τ* value, respectively.

In the case of a very large *τ* (i.e. $\mathrm{1/}\tau \simeq 0$, Fig. 6b), the relaxation dynamics is characterized by a decrease of *w* just after the flux switching off followed by an increase after a transient time; qualitatively confirming the experimental observation. These results can be discussed considering that the mound instability on the scale of *l*_0_, with a so big value of *t*_0_, is always active in the system even in the absence of the flux; the nonlinear ES term of the dynamical Eq. (3) strongly affects the relaxation. However, we point out that the asymptotic behavior of *w* is found to be independent on the rate for very large times, consequently the system seems to lose memory of the different starting state incoherently with the observed phenomenology (for a more exhaustive description see the Supplementary Information (SI)).

In the case of a finite *τ* (Fig. 6c), we optimize the calculation of *w* in order to simulate the experimental behavior using different value of *τ* for each deposition rate. In fact, while the values of *w* at the flux switch-off in Fig. 6b and c can be corrected just changing the value of the noise strength *D*, the agreement of the model with experimental behavior of *w* after *t*_*c*_ is improvable only if we operate on *τ*. While the behavior of *λ* and *ξ* does not change so much, the effect of *τ* is able to extend the relaxation tail at larger times and to speed up the simulated evolution of *w* lowering its value. In particular, the flux rate *R* = 0.086 nm/min corresponds to the situation where the dynamics was observed to be fastest and for our simulation to impose the smallest *τ* value indicating how the relaxation process could be easily represented by quite rigid addition of the term 4 in the Eq. 3.

We emphasize that the simulation well reproduces the experimental observations without strong “ad hoc” hypotheses, but we need to introduce the global parameter *τ*, in order to reproduce the saturation effect at long times of *w* depending on the deposition rate *R*. This indicates that the system does not lose spatial coherence with the relaxation accomplishment taking memory of the original state; an effect maybe connected to the presence of an interaction between the islands that we ascribe to the formation of the adhesion layer and related to the distances (*λ* − *ξ*) of the nanodrops at the end of the growth process.

In the observed phenomenology, we suppose that the side fluoro-alkylic chains of the molecule can play a key role in determining the molecular mobility. As recently demonstrated for a parent compound by friction force microscopy measurements, the side alkylic chains pack into a compact low-surface-energy overlayer with a periodic heterogeneity of the chains bending properties and subsurface anchoring^48^. The formation of a mesophase can break this periodicity also in our molecular system by strongly modifying the adhesion/cohesion energy ratio and the equilibrium between nematic elasticity and the long-range van der Waals forces and so triggering the observed wetting phenomena^49^. However other mechanisms alike some relaxation of HMDS layer, molecular cross-Linking, glass-like relaxation effects or isotropic-nematic phase transitions cannot be excluded.

## Conclusion

In this paper, we analyze the dynamic effect of the critical wetting of PDIF-CN_2_ nano-structured thin-films deposited at room temperature by SuMBD on hydrophobic silicon dioxide. Atomic force microscopy is used to acquire surface morphology monitoring post-deposition transition from partial wetting to a complete wetting during one year. The images were analyzed extracting the characteristic morphological lengths (*w*, *ξ* and *λ*). In parallel, a continuum-equation approach is used to simulate both the growth and post-deposition relaxation. In order to classify the growth, scaling exponents were derived from both the experimental data and the simulations and we observe a slight deviation from typical values of mounded structures. The simulations are in good agreement with the experimental results and the proposed model can be useful, in the framework of the scaling theory, not only to simulate the growth, but also to the description of the solid-on-solid relaxation phenomena and dewetting effects. The need to consider in the non-linear stochastic model a surface equalizer term indicates the presence of a local mechanism of molecular correlation related to the balance between nematic elasticity and long-range van der Waals forces. The change in the molecule-surface interaction likely induces the formation of the adhesion layer on the uncovered part of the surface driving the wetting phenomena.

## Methods

### Material and Thin films Deposition

Every sample has been fabricated via SuMBD deposition from PDIF-CN_2_ powder (Polyera ActivInk N1100) used without any further purification. The organic precursor has been deposited on the top of a square shaped multilayer wafer (1 cm^2^ in area) consisting of a 500 *μ*m thick highly doped silicon substrate, 200 nm thick SiO_2_ layer as insulating material. The dielectric layer has been passivated by means of HMDS self-assembled monolayer via vapor treatment^34^. SuMBD has been performed in a supersonic jet of He carrier gas with stagnation pressure value of 2000 mbar, resulting in kinetic energy values of approximately 17 eV per molecule. Each deposition has been performed with substrates kept at room temperature, while the deposition rate was finely tuned heating the organic powder with temperatures between 203° C and 220° C, corresponding to deposition rates ranging from 0.01 nm/min to 0.22 nm/min. The deposition rate was measured by thickness monitor and after calibrated from the film thickness measurements.

### Characterization

Morphological characterization in air has been performed *ex-situ* by a Park Instruments Xe-100 AFM equipped with Silicon-doped cantilevers, in true-non-contact mode. Statistical analysis of AFM images was accomplished by Gwyddion 2.48 (http://gwyddion.net/). Samples were stored in dry air and in dark.

### Theoretical approach

The height profile and the correlation functions are theoretically evaluated by using a non-linear stochastic continuum equation solved through a mean-field time-dependent self-consistent scheme. All the temporal fluctuations of the non-linear terms are retained, its spatial fluctuations are correctly approximated. The self-consistency is imposed in the momentum space by using the power spectral density function. Very accurate and long dynamics are obtained by means of this approach.

## Electronic supplementary material

Supplementary Information
