# Supplementary material for: Post-Deposition Wetting and Instabilities in Organic Thin Films by Supersonic Molecular Beam Deposition
Source: Sci Rep. 2018 Aug 13;8:12015. doi: 10.1038/s41598-018-30567-7 (PMC6089966; doi:10.1038/s41598-018-30567-7)
Supplement: Supplementary file 1 — Supplementary Information [file 41598_2018_30567_MOESM1_ESM.pdf]

SUPPLEMENTARY INFORMATION
Post-Deposition Wetting and Instabilities in Organic Thin Films by Supersonic Molecular Beam Deposition

F. Chiarella[1,2], C. A. Perroni[2,1], F. Chianese[2,1], M. Barra[1,2],
G. M. De Luca[2,1], V. Cataudella[2,1], A. Cassinese[2,1]
[1] SPIN-CNR Division of Naples, Piazzale Tecchio, 80, I-80125 Naples, Italy.
[2] Physics Department "Ettore Pancini", University of Naples Federico II, I-80125 Naples, Italy.

I. THEORETICAL APPROACH

The focus of the theoretical analysis reported in this supplemental material will be on the step barrier diffusion effect, known also as Ehrlich-Schwoebel (ER) barrier effect. This effect does not allow atoms to diffuse over the edge of a step on the surface creating an overall uphill current of diffusive particle flux. The step barrier diffusion effect creates mounds on the surface, introducing a new length scale λ, which measures the average mound separation. In this study, we will analyze the ER effect not only on the growth process when the particle flux impinges on the substrate, but also on the consequent relaxation dynamics after the flux switching off. In section I, the model and the time-dependent self-consistent approach are reviewed; in section II, the growth in the presence of the flux is theoretically analyzed; in section III, the relaxation dynamics after the flux switching off is discussed.

II. MODEL AND TIME-DEPENDENT SELF-CONSISTENT APPROACH

We will introduce a model for the modeling of the growth dynamics in supersonic molecular beam deposition of PDIFCN2. We will use a local stochastic continuum equation which includes the ER effect. The continuum equation involves the height profile $h(\mathbf{x},t)$ and its derivatives (with respect to the time t and to the space represented by a bi-dimensional vector $\mathbf{x}$) focusing on the local nature of diffusion and the step barrier diffusion effect. Actually, the process of diffusion is localized since one atom/molecule diffuses affecting only its neighbor. Indeed, the ER effect involves the diffusion of particles on the surface, therefore it gives rise to a characteristically local growth.

In this study, the growth process starts from $t = 0$, finishes at t_c, switching off time of the flux, after which a relaxation dynamics takes place. The overall dynamics is modeled by the following equation[1,2] :

$$\frac{\partial h}{\partial t} = -\nu \nabla \cdot \left(\frac{\nabla h}{1 + |\nabla h|^2} \right) - k \nabla^4 h + \eta(\mathbf{x},t) + F(t) + \left(\frac{\partial h}{\partial t} \right)_r , \quad (1)$$

where the first term describes the uphill growth due to the ER effect (∇ is the gradient operator), the second one is the Mullins diffusion term modeling the overall effect of surface diffusion. We remark that in the first term there is the sign $-$, therefore the ER effect introduces an instability in the growth process favoring the formation of mounds. The stochastic term present during the growth is modeled through the noise $\eta(\mathbf{x},t)$, which is, as usual, assumed Gaussian:

$$< \eta(\mathbf{x},t) > = 0,$$
$$< \eta(\mathbf{x},t)\eta(\mathbf{x}',t') > = 2D\delta(\mathbf{x}-\mathbf{x}')f(t)\delta(t-t'), \quad (2)$$

where $f(t) = \theta(t-t_c)$, with $\theta(t)$ Heaviside theta function. In Eq. (1), the quantity $F(t)$ models the particle flux:

$$F(t) = Rf(t), \quad (3)$$

where R is the flux rate. The last term of Eq. (1) provides a characteristic relaxation time τ:

$$\left(\frac{\partial h}{\partial t} \right)_r = -\frac{[h(\mathbf{x},t) - G(t)]}{\tau}, \quad (4)$$

with $G(t)$ indicating a primitive of the flux term

$$G(t) = \int_0^t dt_1 F(t_1) = Rt_c. \quad (5)$$

This last term will be important after the switching off of the flux impinging on the substrate. The dynamics described by Eq. (1) is nonlinear due to the ER first term. Actually, the non-linear contribution in the denominator of the first term allows to provide a limit to the instability related to the mounding.

We derive the temporal behavior of all the relevant dynamical quantities by using a time-dependent self-consistent approach. The starting point is the observation that

$$< |\nabla h|^2 > = m^2(t), \quad (6)$$

where $m(t)$ is the surface slope. In order to solve the stochastic equation, we retain all the temporal fluctuations of the non-linear contribution in the first term of Eq. (1), approximating its spatial fluctuations:

$$\nabla \cdot \left(\frac{\nabla h}{1 + |\nabla h|^2} \right) \simeq \frac{\nabla^2 h}{[1 + m^2(t)]}. \quad (7)$$

Therefore, in the dynamical equation, one gets a term which depends itself on the equation solution. Therefore, we will solve the equation by using a self-consistent

approach: the solution $h(\mathbf{x},t)$ is accepted only when the slope function $m(t)$ in the equation is, within a fixed tolerance, equal to that directly calculated through the height profile.

In order to manage the inhomogeneous terms of Eq. (1), we divide the height profile into two contributions:

$$h(\mathbf{x},t) = h'(\mathbf{x},t) + G(t), \tag{8}$$

where $h'(\mathbf{x},t)$ is the height profile affected by the stochastic noise, while $G(t) = Rt_c$ is the temporal contribution due to the external flux. We point out that $G(t) = Rt_c$ represents the average thickness of the organic film. The profile $h'(\mathbf{x},t)$ satisfies the following stochastic equation:

$$\frac{\partial h'}{\partial t} = -\nu \frac{\nabla^2 h'}{[1+m^2(t)]} - k\nabla^4 h' + \eta(\mathbf{x},t) - \frac{h'}{\tau}, \tag{9}$$

with $< |\nabla h|^2 >=< |\nabla h'|^2 >= m^2(t)$. In addition to the slope $m(t)$, all the relevant quantities can be calculated through the height profile h': the interface width $w(t)$ defined as

$$< |h'|^2 >= w^2(t), \tag{10}$$

the autocorrelation function $R(\mathbf{r},t)$ as

$$R(\mathbf{r},t) = \frac{< h'(\mathbf{r}+\mathbf{x},t)h'(\mathbf{x},t) >}{w^2(t)}, \tag{11}$$

so that the height-height correlation function $H(\mathbf{r},t)$ is

$$H(\mathbf{r},t) = \left\langle [h'(\mathbf{r}+\mathbf{x},t) - h'(\mathbf{x},t)]^2 \right\rangle = 2w^2(t)[1-R(\mathbf{r},t)]. \tag{12}$$

We solve Eq. (9) in the bi-dimensional momentum space $\mathbf{q}$, therefore, we consider the spatial Fourier transform $\hat{h}(\mathbf{q},t)$ of $h'(\mathbf{x},t)$. One gets the following temporal equation for $\hat{h}(\mathbf{q},t)$:

$$\frac{\partial \hat{h}(\mathbf{q},t)}{\partial t} = \left(\frac{\nu q^2}{[1+m^2(t)]} - kq^4 - \frac{1}{\tau} \right) \hat{h}(\mathbf{q},t) + \hat{\theta}(\mathbf{q},t), \tag{13}$$

with $q = |\mathbf{q}|$ is the modulus of the vector $\mathbf{q}$, and $\hat{\theta}(\mathbf{q},t)$ is the spatial Fourier transform of the Gaussian noise term $\eta(\mathbf{x},t)$, therefore

$$< \hat{\theta}(\mathbf{q},t) > = 0,$$
$$< \hat{\theta}(\mathbf{q},t)\hat{\theta}(\mathbf{q}',t') > = 2D\delta(\mathbf{q}+\mathbf{q}')f(t)\delta(t-t'). \tag{14}$$

The first-order temporal non-linear equation can be analytically solved determining the power spectral density function $P(\mathbf{q},t)$, defined as

$$P(\mathbf{q},t) =< |\hat{h}(\mathbf{q},t)|^2 > . \tag{15}$$

We stress that the density function $P(\mathbf{q},t)$ is proportional to D, the strength of the noise. Moreover, this density function allows to calculate all the relevant quantities of the dynamics. Actually, the function $P(\mathbf{q},t)$ is the spatial Fourier transform of the autocorrelation function $R(\mathbf{r},t)$ given in Eq. (11). Furthermore, it allows to directly calculate the interface width $w(t)$ being

$$w^2(t) = \int d\mathbf{q} P(\mathbf{q},t). \tag{16}$$

Finally, we point out that the power spectral density function $P(\mathbf{q},t)$ depends on the slope $m(t)$ being derived from the solution of Eq. (13), but, at the same time, it allows to derive the slope:

$$m^2(t) = \int d\mathbf{q} |\vec{q}|^2 P(\mathbf{q},t). \tag{17}$$

This turns out to be the equation that we have self-consistently solved to determine the actual density function. Indeed, at the zero order, we have fixed $m_0(t) = 0$, then, form Eq. (17), we get $m_1(t)$, and so on. A few steps of iteration are necessary to get a function $m_i(t)$ very close to $m_{i+1}(t)$ (a tolerance is given in the norm of the difference between $m_i(t)$ and $m_{i+1}(t)$). It is the behavior at long times which is progressively improved with increasing the iterative steps. We stress that the self-consistent approach allows to carefully analyze the solutions for very long times.

Most organic films analyzed in the experiments have an average thickness T around 20 nm. Since the flux rate R used in the experiments is known, one can estimate the switching off time $t_c = T/R$. The rates R used in the experiments vary from around 0.04 nm/min to 0.22 nm/min. As a consequence, the times t_c derived from experiments go from around 90 min to 500 min.

With the parameters ν and k present in Eq. (13), one can obtain the length $l_0 = 1/q_0 = \sqrt{2k/\nu}$ and the time $t_0 = l_0^3/\sqrt{\nu k} = 2\sqrt{2}k/\nu^2$. The quantities l_0 and t_0 provide a term $D_0 = l_0^4/t_0 = \sqrt{2}k$, which has the same dimensions as the term D, which measures the strength of noise. For the dynamics analyzed in this paper, we set the following values of l_0 and t_0 as unit length and time, respectively: $l_0 = 77$ nm and $t_0 = 1.38$ days. Then, the free parameters of the theory are R (or t_c), D, given in terms of D_0, τ, expressed in terms of t_0. For the dynamics analyzed in this paper, D is of the order of $0.001 - 0.01$ D_0, while the relaxation time τ is assumed to be quite large being of the order of $6 - 7$ t_0.

III. GROWTH IN THE PRESENCE OF THE FLUX

In this section, we analyze the growth process in the presence of a flux impinging on the substrate, therefore in the case of times $t < t_c$. We will analyze the growth features for t_c larger than 500 min. Since the values of the relaxation τ are assumed large, the effects of this parameter on the growth dynamics are completely negligible for times $t < t_c$.

We will investigate the interface width $w(t)$ focusing in particular on the regime of power-law behavior and the related exponent β in the presence of the flux:

$$w(t) \sim t^{\beta}. \tag{18}$$

Another important quantity is the lateral correlation length $\xi(t)$ defined through the autocorrelation function of Eq. (11) such that

$$R(\xi(t), t) = 1/e \simeq 0.36787. \tag{19}$$

We will study the correlation length $\xi(t)$ focusing in particular on the exponent $1/z$ which characterizes the regime of power-law behavior in the presence of the flux:

$$\xi(t) \sim t^{1/z}. \tag{20}$$

In the case of mounding, another important length is the average mound separation $\lambda(t)$, which plays the role of the characteristic wave-length. At a fixed time t, given the width $w(t)$, the length $\lambda(t)$ is determined assuming, in the limit of large distance ($|\mathbf{r}| >> \xi(t)$), the following behavior of the height-height correlation function of Eq.(12):

$$H(|\mathbf{r}|, t) \simeq 2w^2(t) J_0 \left(\frac{2\pi|\mathbf{r}|}{\lambda(t)} \right), \tag{21}$$

where $J_0(x)$ is the zero-order Bessel function. We will analyze $\lambda(t)$ focusing in particular on the exponent p which characterizes the regime of power-law behavior in the presence of the flux:

$$\lambda(t) \sim t^{p}. \tag{22}$$

When the mound instability is absent, the self-affine behavior takes place and the dynamic scaling is present, that is $p = 1/z$. In general, dynamic scaling does not hold for mounded surfaces. In the following, we will find slight deviations from the situation of dynamic scaling.

In Fig. 1, we show the average mound separation λ, the lateral correlation length ξ, and the interface width w as a function of time. At first, we notice the different orders of magnitude of these lengths. Actually, the width w is of the order of a few nm, the correlation length ξ is of the order of ten nm, the wave-length λ of the order of one hundred nm. We stress that, in case of mounding instability, the correlation length ξ provides the average size of a mound. Therefore, one expects that ξ is smaller than λ.

In the presence of a flux, after a short transient, all the three lengths follow a power-law t^{γ}. We have fitted all the three time functions in the experimentally relevant range of t_c, therefore, as shown in Fig. 1, we consider a time interval between 1 hour and 6.5 hours. The exponent β of the interface width is about 0.29, a value perfectly compatible with that found in the literature neglecting the correction $m^2(t)$ in the ER term of the dynamical equation[3]. This result is expected since, up

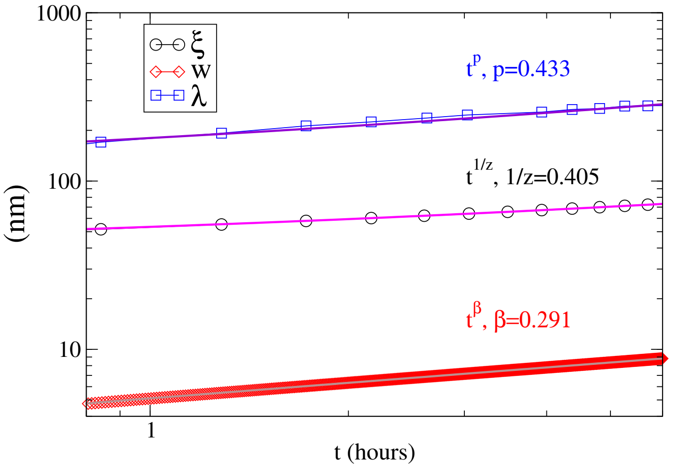

FIG. 1. Average mound separation λ, lateral correlation length ξ, and interface width w (in units of nm) as a function of time (in units of hours). In the plot, $D = 0.005$ (in units of D_0), $1/\tau \simeq 0$.

to times of the order of a few hours, the values of $m^2(t)$ are always smaller than 1. Therefore, the self-consistent procedure provides weak perturbations when the flux is present. The other two lengths increase more quickly as a function of times. Actually, the exponent p of λ is about 0.433, while the exponent $1/z$ of the correlation length ξ is around 0.405. Therefore, the dynamics scaling is only weakly violated. In the main text, we plot the correlation length ξ and the mound separation λ as a function of the flux rate making a comparison with experimental data.

IV. RELAXATION AFTER THE FLUX SWITCHING OFF

In this section, we analyze the relaxation dynamics from the flux switching off up to times larger than 20 days. Our self-consistent procedure is able to cover this very large time range providing accurate numerical results. We stress that the slope correction $m^2(t)$ in the ER term of the dynamical equation strongly affects the relaxation. First, we will consider the case of an infinite τ ($1/\tau \simeq 0$), then the effects of a finite τ. We will show that a large but finite τ plays an important role in order to interpret the dynamical relaxation.

In Fig. 2, we show the lateral correlation length ξ as a function of time for different flux rates in the case $1/\tau \simeq 0$. We theoretically analyze the cases of intermediate ($R = 0.1$ nm/min), very large ($R = 1$ nm/min) and very small ($R = 0.01$ nm/min) flux rate. Focus is on the relaxation dynamics, therefore we plot the behavior of ξ only for $t \geq t_c$. We find that the asymptotic behavior of ξ at very large times is independent of the rate. Moreover,

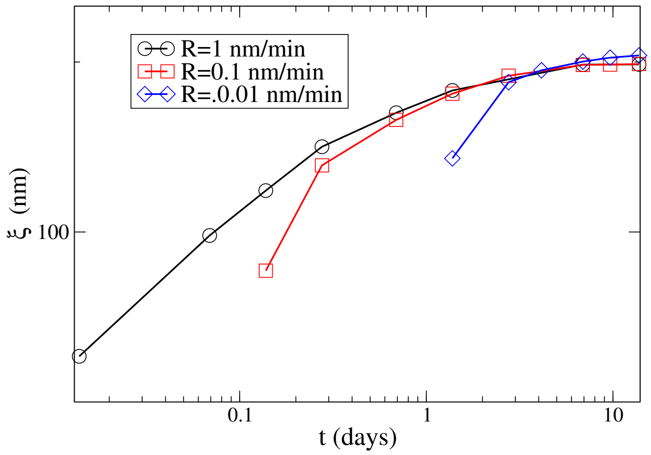

FIG. 2. Lateral correlation length ξ (in units of nm) as a function of time t (in units of days) for different values of the flux rate R (in units of nm/min). In the plot, $D = 0.005$ (in units of D_0), $1/\tau \simeq 0$.

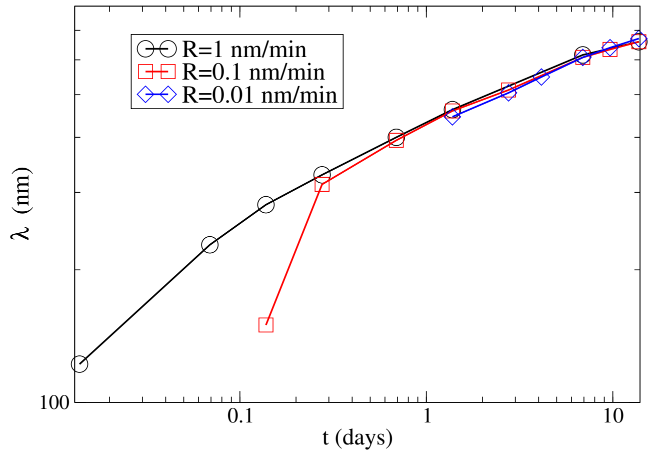

FIG. 3. Average mound separation λ (in units of nm) as a function of time t (in units of days) for different values of the flux rate R (in units of nm/min). In the plot, $D = 0.005$ (in units of D_0), $1/\tau \simeq 0$.

at very large times, ξ increases slowly reaching a nearly constant asymptotic value close to 200 nm. Therefore, as a result of the relaxation, the average size of a mound can be a stable quantity as a function of time.

In Fig. 3, we show the average mound separation λ as a function of time for different flux rates in the case $1/\tau \simeq 0$. As in the case of the correlation length, we theoretically analyze the cases of intermediate ($R = 0.1$ nm/min), very large ($R = 1$ nm/min) and very small ($R = 0.01$ nm/min) flux rate for $t \geq t_c$. In analogy with ξ, the asymptotic behavior of λ is poorly independent of the rate at very large times. In contrast with ξ, at very large times, λ increases as a function of time even if the increase is quite slow. Therefore, during the relaxation, the average mound separation is a slow function of time. One can expect such a behavior since the mound instability is always active in the system even in the absence of the flux.

We point out that the values of λ for times around 20 days are of the order of 650 nm, a value that is not much larger than $2\pi/q_0 = 2\pi l_0 = 2\pi\sqrt{2k/\nu} \simeq 480$ nm. This last value represents the natural average mound separation in a linear equation model without the corrective term $m^2(t)$ in the ER term[3]. We can interpret the numerical results considering that, due the slope corrective term, there is an effective $\nu_{eff} \simeq \nu/(1 + m^2_{eff})$, where m_{eff} is an effective value determined by the type and duration of the dynamics. Therefore, one expects a smaller ν_{eff}, therefore a larger $\lambda_{eff} \propto \sqrt{1/\nu_{eff}}$.

The increase of the values of λ as a function of time is obtained by analyzing the behavior of the height-height correlation function in the limit of large spatial separation. In Fig. 4, we show the height-height correlation

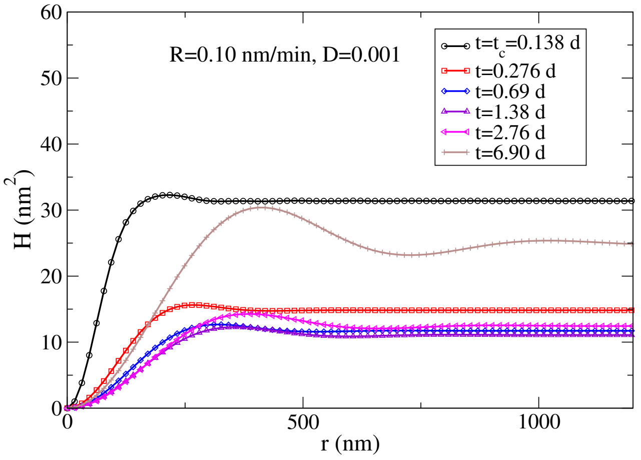

FIG. 4. Height-height correlation function (in units of nm^2) as a function of distance $r = |\mathbf{r}|$ (in units of nm) for different values of the time t (in units of days d). In the plot, $D = 0.001$ (in units of D_0), $1/\tau \simeq 0$, R=0.10 nm/min.

function as a function of distance for different values of the time t at an intermediate value of the flux rate R ($R = 0.1$ nm/min). It is apparent that the oscillatory behavior for large distances is also more marked with increasing time. Moreover, we notice that, just after the flux switching off time, the values of the correlation function get reduced. Only after a transient time of the order of a few days, the values of H starts increasing. We emphasize that this behavior is in good agreement with

experimental data. This trend can be almost entirely ascribed to the time behavior the width $w(t)$ (shown in the main text) being the correlation function H proportional to $w^2(t)$. As shown in the main text, the effect of a finite τ reduces $w(t)$ at large times, therefore it will further decrease the values of the correlation function H at intermediate flux rate, getting a better agreement with experimental data.

[1] M. Pelliccione and T.-M. Lu, *Evolution of Thin Film Morphology - Modeling and Simulations* (Springer Series in Materials Science, 2008).
[2] A.-L. Barabasi, H. E. Stanley, *Fractal Concepts in Surface Growth* (Cambridge University Press, 1995).
[3] Y.-P. Zhao, H.-N. Yang, G.-C. Wang, and T.-M. Lu, Phys. Rev. B **57**, 1922 (1998).
